# Lymph Node Metastasis From Gastroesophageal Cancer Successfully Treated by Nivolumab: A Case Report of a Young Patient

**DOI:** 10.3389/fonc.2019.01375

**Published:** 2019-12-16

**Authors:** Shin Kashima, Hiroki Tanabe, Mishie Tanino, Yu Kobayashi, Yuki Murakami, Takuya Iwama, Takahiro Sasaki, Takehito Kunogi, Keitaro Takahashi, Katsuyoshi Ando, Nobuhiro Ueno, Kentaro Moriichi, Masahide Fukudo, Yoshikazu Tasaki, Masao Hosokawa, Yusuke Mizukami, Mikihiro Fujiya, Toshikatsu Okumura

**Affiliations:** ^1^Division of Gastroenterology and Hematology/Oncology, Department of Medicine, Asahikawa Medical University, Asahikawa, Japan; ^2^Department of Surgical Pathology, Asahikawa Medical University, Asahikawa, Japan; ^3^Department of Hospital Pharmacy and Pharmacology, Asahikawa Medical University, Asahikawa, Japan; ^4^Department of Surgery, Keiyukai Sapporo Hospital, Sapporo, Japan

**Keywords:** gastric cancer, immunotherapy, complete remission (CR), tumor burden, lymph node metastasis (LNM)

## Abstract

**Background:** Immuno-oncology is a novel target of cancer therapy. Nivolumab is a monoclonal anti-programed death-1 antibody recently used to treat patients with chemotherapy-resistant gastric and gastroesophageal cancer. Although the disease control rate is reported to be very high, few cases demonstrate a complete response.

**Case Presentation:** A 25-year-old man diagnosed with gastroesophageal cancer was treated with chemotherapy followed by surgical resection. Pathological diagnosis was poorly differentiated adenocarcinoma with distant lymph node metastasis. Residual lymph node metastasis was treated with nivolumab monotherapy, resulting in complete disappearance. No recurrence has been observed for 2 years since discontinuation of nivolumab. This rare case was additionally subjected to pathological and genetic analysis, suggesting that a high tumor mutation burden (10.7 mutations/Mb) might be associated with sensitivity to nivolumab.

**Summary:** We reported a case of advanced gastroesophageal junction cancer with distal lymph node metastasis that was successfully treated with chemotherapy, surgical resection, and nivolumab therapy. An aggressive search for biomarkers implying benefit effects of nivolumab should be performed.

## Summary

We encountered a case of gastroesophageal cancer case with a complete response to nivolumab therapy in a young patient who possessed a high mutation burden and germ-line TP53 SNP. Further genetic and histological analyses will help exploring biomarkers.

## Introduction

Cancer immunotherapies targeting immunosuppressive checkpoint receptors have dramatically changed the strategy of cancer therapy. The pathway between programmed death-1 (PD-1) and its ligand programed death-1 ligand 1 (PD-L1) is a target of immuno-oncology, and anti PD-1 monoclonal antibodies have been used to treat chemotherapy-refractory cancer patients. Nivolumab and pembrolizumab were approved by the FDA in 2014 and have been used for the treatment of melanoma, non-small cell lung cancer, melanoma, and renal cell carcinoma (1, 2). Clinical studies have been extensively performed in order to expand their clinical application to other types of carcinoma (3).

Gastric cancer is the fifth-most common cancer and the third leading cause of cancer death, worldwide (4). However, more-effective therapies are awaited and immune-oncology is expected to provide a cure for chemotherapy-refractory cancer patients. A randomized controlled trial with nivolumab or a placebo in patients with advanced gastric or gastro-esophageal junction cancer (ATTRACTION-2) suggested that nivolumab might be a treatment option for pretreated patients (5). Among the study population of 493 patients, 226 were enrolled from Japanese institutions. A subpopulation analysis of the Japanese patients showed that the overall survival of the nivolumab group was longer, the risk of death lower, and serious adverse events rarer than in the placebo control group (6). Concerning the best overall responses, the rates of complete response, partial response, and stable disease were 0, 14.0, and 31.0%, respectively. The disease control rate was as high as 45.0%, although there were no cases reaching complete response.

We herein report a case of advanced gastroesophageal junction cancer with distal lymph node metastasis that was successfully treated with chemotherapy, surgical resection, and nivolumab therapy.

## Case Presentation

A 25-year-old man complained of dysphagia and epigastric pain for 2 months and underwent esophagogastroduodenoscopy (EGD) at a gastrointestinal clinic, where a large tumor at the gastroesophageal junction was detected. He was referred to our hospital for the further diagnosis and therapy.

**Figure 1:**
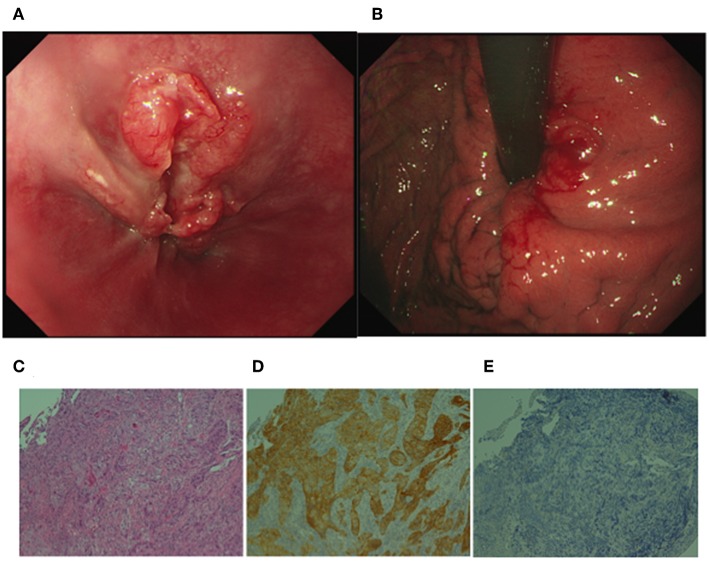
Upper gastrointestinal endoscopic images and histological analyses of the gastroesophageal junction cancer. **(A)** Deep ulceration with a rounded bank was observed in the junction. **(B)** Swelling of the gastric fold indicated submucosal invasion of the cancer cells in the cardia of the stomach. **(C)** H&E staining of the biopsy specimen showed poorly differentiated adenocarcinoma. **(D)** Immunohistochemistry revealed that gastric cancer cells were positive for human epidermal growth factor 2 (HER2). **(E)** An SP142 assay revealed that both cancer cells and stromal cells were negative for programmed death-1 ligand 1. (**C–E** original magnification ×200).

**Figure 2:**
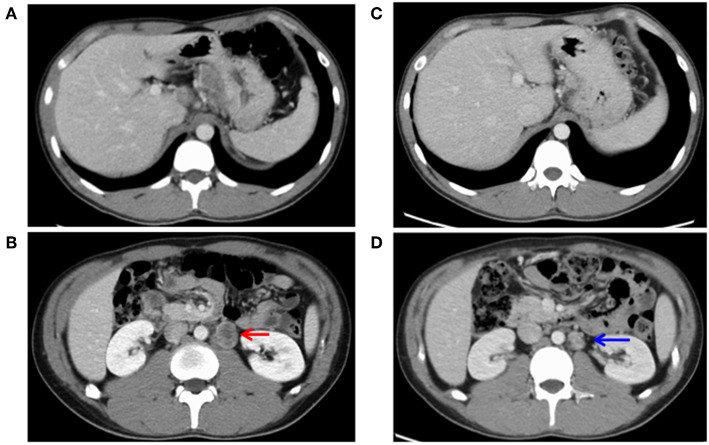
Images of computed tomography before **(A,B)** and after **(C,D)** chemotherapy. **(A)** Cardiac wall thickness and connected lymph node swelling was observed. **(B)** Paraaortic lymph node metastasis (#16) showed distant metastasis of 30 mm in size (indicated by a red arrow). **(C)** The lymph node of the gastric cardia had shrunk. **(D)** The paraaortic lymph node metastasis (#16) had shurunk to 19 mm in size (a blue arrow), showing a partial response.

He had no specific medical history or family history of gastric malignancy. Laboratory examination presented normal blood counts (white blood cells, 4.63 × 10^3^/mm^3^; red blood cells, 4.93 × 10^6^/mm^3^; platelets, 197 × 10^3^/mm^3^), and the neutrophil to lymphocyte ratio (NLR) was 3.82 (neutrophils, 3.44 × 10^3^/mm^3^; lymphocytes, 0.90 × 10^3^/mm^3^). No significant findings were observed in blood chemistry [e.g., lactose dehydrogenase (LDH), 167 IU/l]. But tumor markers were elevated [carcinoembryonic antigen (CEA), 1215.0 ng/ml; alpha fetoprotein (AFP), 546.0 ng/ml]. A physical examination upon admission showed no abdominal tenderness or superficial lymph node swelling. The performance status developed by Eastern Cooperative Oncology Group was grade 0. Advanced cancer was noted at the gastroesophageal junction (Figures 1A, B), and a biopsy specimen showed poorly differentiated adenocarcinoma (Figure 1C). An immunohistochemical analysis was performed using a BOND-III autoimmunostaining system (Leica Biosystems, Nussloch, Germany). Immunostaining revealed that the gastric cancer cells (determined with clone CB11) were strongly positive for human epidermal growth factor 2 (HER2) (Figure 1D). Both cancer cells and stromal cells were negative for PD-L1 [determined with clones SP142 and SP263 [Roche, Basel, Switzerland]] (Figure 1E). Computed tomography (CT) showed wall thickness in the cardia of the stomach and swelling of multiple abdominal lymph nodes including the paraaortic #16 lymph node (Figures 2A,B). The clinical diagnosis was stage IV gastroesophageal junction carcinoma.

**Figure 3:**
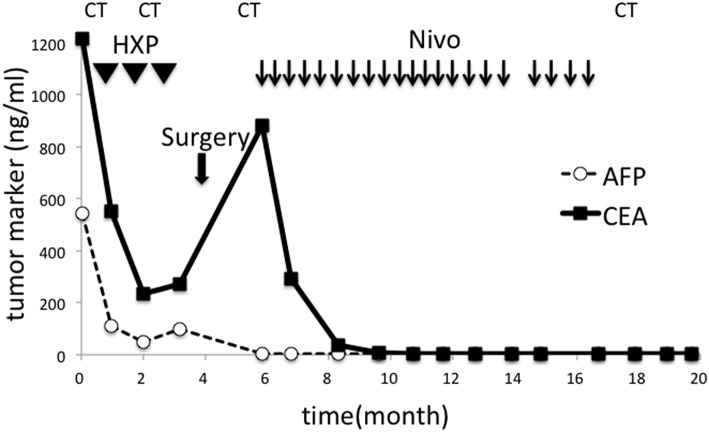
Changes in tumor markers during the course of the treatment. CEA and AFP sharply decreased after chemotherapy but re-elevated after three cycles. Surgery failed to reduce CEA, but nivolumab was effective. Tumor markers remained in their normal ranges after discontinuation of all treatment. HXP, Herceptine, Xeloda, and Cisplatin; Nivo, nivolumab; CT, computed tomography.

Surgical resection was not performed. Instead, chemotherapy with herceptin (8 mg/kg on day 1), capeciabine (2,000 mg/m^2^ day 1–14), and cysplatin (80 mg/m^2^ on day 1) was conducted as the first-line treatment. After three cycles, CT showed a partial response in the paraaortic lymph node (Figures 2C,D), but EGD showed no change in the main lesion. Decreased tumor markers were re-elevated (Figure 3), so we decided to change the treatment strategy. Surgical resection was selected, and radical total gastrectomy with paraaortic lymph node dissection was performed. The pathological diagnosis was type 2 carcinoma at the gastroesophageal junction, poorly differentiated adenocarcinoma pT3 (SS), int, ly3, v2, N3b (22/94), M1 LYM, Stage IV according to Japanese Classification of Gastric Carcinoma (The 15th Edition).

**Figure 4:**
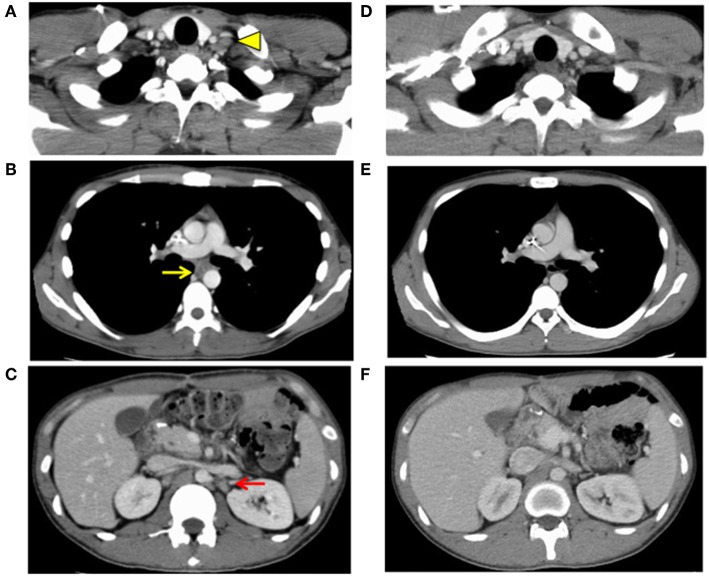
Images of computed tomography 1 month after surgery **(A–C)** and after finish of the nivolumab threatment **(D–F)**. Lymph node metastasis was observed **(A)** in the subclavicle (#104L, yellow arrowhead), **(B)** mediastinum (#108L, yellow arrow), and **(C)** paraaorta (#16b1, red arrow), indicating distally metastatic recurrence of the carcinoma (progressive disease). Lymph node metastasis of **(D)** the subclavicle (#104L), **(E)** mediastinum (#108L), and **(F)** paraaorta (#16b1) had vanished, indicating a complete response.

One month after surgery, CT showed lymphadenopathy in the mediastinum (#108L), subclavicle (#104L), and abdomen (#11p, #16b1) (Figures 4A–C). Nivolumab treatment (3 mg/kg every 2 weeks) was conducted as the secondary chemotherapy. The NLRs were 0.92 (neutrophils, 1.02 × 10^3^/mm^3^; lymphocytes, 1.10 × 10^3^/mm^3^) and 0.57 (neutrophils, 0.69 × 10^3^/mm^3^; lymphocytes, 1.21 × 10^3^/mm^3^) before and a week after first nivolumab injection, respectively. The lymph node metastasis was dramatically shrunk after four cycles of Nivolumab therapy and ultimately disappeared. Nivolumab monotherapy was stopped at 48 weeks because the tumor had completely diminished after 21 cycles (Figures 4D–F). No recurrence has been observed for 2 years since the discontinuation of any treatments.

### Pathological Analyses (Supplementary Figure 1)

An examination of the surgically resected tissue showed that the gastric cancer cells infiltrating the subserosa were still alive even after chemotherapy. Lymphocytes did not massively infiltrate into the tumor, nor did they stain specifically for CD8, CD4, FoxP3, or PD-1 antibodies.

An immunohistochemical study showed that mismatch-repair (MMR) proteins of mutL homolog 1 (MLH1), mutS homolog 2 (MSH2), mutS homolog 6 (MSH6), and postmeiotic segregation increase 2 (PMS2), had not disappeared in the gastric cancer tissue of the surgically resected stomach. In our assessment of the association with Epstain-Barr virus (EBV), EBV-encoded RNA *in situ* hybridization showed negative staining. PD-L1 expression is a predictive marker for responders to PD-1 inhibitors, so the PD-L1 expression was investigated, showing hypo-expression in tumor cells and immune cells. Similar staining patterns were observed in the gastric cancer cells of the dissected paraaortic lymph node (#16).

### Genetic Analyses (Supplementary Figure 2)

Microsatellite instability (MSI) was determined using a kit (MSI analysis system v1.2, Promega, Madison, WI) according to the manufacturer's instruction. There was no shift in the peak of macrosatellite markers on comparing the cancerous and normal tissue, indicating microsatellite stability (MSS). This result confirmed the immunohistochemistry of MMR proteins (MMR-proficient). Genomic mutations and variants were tested according to previously described methods (7). The mutation rate was 10.74 mutations per Mb, with 5.37 non-synonymous mutations per Mb, which was considered with a hyper-mutated status. Detailed mutation data are shown in Supplementary Data Sheet 1.

A single nucleotide polymorphism (SNP) was found in TP53 c.215C>G, p.Pro72Arg (P72R), which was deposited as a Japanese SNP.

### Concentration of Nivolumab

Trough concentrations in the serum of the patient measured using an in-house enzyme-linked immunosorbent assay (8), were 56.3 and 63.8 μg/ml at cycles of 17 and 19, respectively. The concentrations were within normal ranges (9), as determined by our institute.

## Comments

We presented a very rare case of gastroesophageal junction cancer that completely responded to Nivolumab. This approach of sequential treatment with chemotherapy, surgical resection, and immunotherapy was dramatically successful in our patient. PD-1 checkpoint inhibition with Nivolumab has become a standard treatment for the patients with advanced gastric carcinoma who are resistant to cytotoxic chemotherapy (10).

The mechanism of action and clinical efficacy of anti PD-1 therapies have been extensively studied and reviewed elsewhere (11, 12). The PD-1 pathway contributes to the regulation of immunological tolerance, and the blockage of the pathway thus restores the immune response to tumor cells. Nivolumab was approved for the treatment of gastric cancer as well as melanoma, lung cancer and renal cell carcinoma. The clinical effectiveness has also been proved against other types of cancers, such as bladder cancer, Hodgkin's lymphoma, and head and neck cancer (13). However, nivolumab is effective in only some patients with cancers in which its clinical use is permitted. Therefore, predictive biomarkers are needed for the patient selection and for making decisions on treatment continuation. Clinical, blood, and tissue biomarkers have been studied in relation to immune-checkpoint inhibitors (14).

Our patient was young enough to show good performance status with normal blood test results, with the exception of high tumor marker levels. It was curious that NLR was very high at the primary admission and became lower while the immunotherapy. Blood parameters such as the neutrophil and lymphocyte counts, and the NLR are easily and repeatedly tested and are therefore recommended as standard markers for patients treated with chemotherapy (15). The serum LDH levels have been reported to correlate with overall survival in various treatments. These markers have been frequently reported to be prognostic values but their role as predictive markers in immunotherapy still under discussion (16).

Immune biomarkers are candidates that should be explored for assessing the response to immune checkpoint therapies (17, 18). A dominant mechanism in the blockade of PD-1/PD-L1 interaction by anti-PD-1 drugs is associated with the PD-L1 expression in tumor cells. Performing evaluations based on immunohistochemistry may help predict the anti-PD-1 therapy response, and such an analysis was performed in the study of nivolumab in 39 patients with several solid tumor cell types (8). Since that initial report, the findings have been validated in investigations performed in several other solid tumors, such as lung cell cancer, melanoma, renal cell cancer, and bladder cancer, using a number of different PD-L1 immunohistochemistry assays and cut-off criteria for positivity (19–21). The tumor proportion score (TPS) is usually used as an indicator of the PD-L1 expression but the expression in immune cells is also used as an indicator of the cut-off value (22). In the phase 3 trial of nivolumab for gastric cancer, The PD-L1 expression status (1% TPS) showed no marked differences in the median overall survival among the subpopulations (5). Even in patients with PD-L1-negative tumors (TPS < 1%), the median overall survival was 6.05 months in the nivolumab group and 4.19 months in the placebo group, showing a statistical benefit of nivolumab. Ongoing analyses concerning the cut-off value of the PD-L1 expression are still in progress. There are some other concerns in relation to the histological evaluations, since biopsy and surgically resected specimens or primary tumors and metastatic lesions showed different expression levels (23, 24).

Intratumoral lymphoid infiltration in different tumor types is reported to improve the prognosis (25). The presence of tumor-infiltrating lymphocytes (TILs) has a prognostic potential for each tumor. T-cell recruitment and the coordinated local adaptive antitumor immune response may be highly organized. CD8+ T cell density at the invasive tumor edge is shown to correlate with the response to anti-PD-1 treatment.

Genetic biomarkers, such as oncogenic mutations and DNA MMR complex, have also been extensively examined (26). The prevalence of somatic mutations across human cancer types was examined in the Cancer Genome Project (27). Genetic mutations have the capacity to encode neoantigens that are specific to the tumor relative to normal somatic cells. Cancer mutations can produce neoantigenic peptides via multiple mechanisms. T-cells recognize the processed peptides presented by major histocompatibility complex (MHC) molecules. A high mutation load might predict responsiveness to immune checkpoint blockade and the clinical observation that cancers with the highest high mutation load (e.g., melanoma or lung cell cancer) showed a high response rate to anti-PD-1 therapy confirmed this theory. The tumor mutation burden is increased by a specific genetic subset associated with multiple cancer types. A subset is defined by defects in the DNA MMR complex. This group of genes was originally discovered in a case of familial cancer syndrome termed Lynch syndrome (28). A clinical trial showed that deficiency in MMR (MMR-d) or MSI-high is a predictive marker for response to PD-1 blockade in advanced cancer patients (29).

Virus-associated cancers may incorporate genetic alterations in cancers, including EBV, human papilloma virus, human T-lymphotropic virus, and hepatitis B virus. Gastric cancer is classified into four groups in the Cancer Genome Atlas; EBV-positive, MSI, chromosomal instability, and genomically stable. EBV-infected gastric cancers are frequently positive in PD-L1 immunohistochemistry (30). PD-1 blockade may therefore induce a greater response in EBV-positive gastric cancers. Clinical trials to test the efficacy of PD-1 pathway blockade in virus-associated cancers are underway at present.

Genetic and immunohistochemical analyses should be performed for the selection of patients in whom treatments will be most effective. Our case had MSS according to an MSI analysis and was not deficient for MMR proteins of MLH1, MSH2, MSH6, and PMS2. EBV *in-situ* hybridization was negative, and TILs were not observed in the surgically resected gastric specimen obtained before anti PD-1 therapy. In the analysis with next-generation sequencing, this case showed a mutation burden of 10.74 mutations per Mb, which was deemed hyper-mutated (26). The genetic variants, TP53Q104^*^ and BRCA1 E1754G were found in the gastric carcinoma, but these variants have not been reported to have any association with PD-1 therapy.

The germline mutations of TP53 usually have important clinical implications as Li-Fraumeni syndrome. The present patient was a very young Japanese man with gastroesophageal cancer, and therefore his disease was suspected to be related to familial germline mutations of oncogenes. TP53 P72R polymorphism was observed, but the relevance of P72R for cancer susceptibility remains dubious. Codon 72 is located within a proline-rich region and may affect a putative SH3-binding domain (31). Many studies have reached the current consensus that R72 is more effective in inducing apoptosis than P72 (32, 33). As such, the P72R mutation may be associated with the sensitivity to nivolumab therapy and not with tumor initiation.

More analyses of clinical responders to anti PD-1 treatment with next-generation sequencing studies as well as histochemical analyses are still needed. The numbers of cases of malignant melanoma and non-small cell lung carcinoma effectively treated by nivolumab have been increasing steadily, and candidate biomarkers are being explored. To our knowledge, complete responders are still rare in gastric cancers, although a case report of liver metastasis from gastric cancer described the achievement of a complete response by nivolumab therapy (34). However, the authors did not present any reason for the remarkable response. Genetic and immunohistochemical analyses with further clinical application will be needed to tailor sensitive treatments to suitable patients. Searches for biomarkers indicating the clinical benefit of nivolumab treatment should be continued. The present report was associated with a limitation. Specifically, the tissue from distal metastatic lymph nodes was not tested in our genetic and histochemical analyses. The target lesion of the nivolumab treatment was recurrent lymph node metastasis after surgical resection, and had been eliminated for 2 years.

## Data Availability Statement

All datasets for this study are included in the article/Supplementary Material.

## Ethics Statement

Written informed consent was obtained from the patient of this case presentation. The treatment for the patients was approved by Asahikawa Medical University Ethics Committee (No. 16190).

## Author Contributions

SK designed the entire treatment. HT wrote the manuscript. MT was in charge of pathological diagnosis and histological study. YK, YMu, TI, TS, TK, and KT were involved in the treatment of the patients. KA and NU carried out genetic analysis. KM was in charge of genetic sequencing. MFuk and YT were involved in the measurement of Nivolimab concentrations. MH was involved in surgical treatment. YMi, MFuj, and TO reviewed the manuscript. All authors approved the final manuscript.

### Conflict of Interest

The authors declare that the research was conducted in the absence of any commercial or financial relationships that could be construed as a potential conflict of interest.
