# Supplementary material for: Lymph Node Metastasis From Gastroesophageal Cancer Successfully Treated by Nivolumab: A Case Report of a Young Patient
Source: Front Oncol. 2019 Dec 16;9:1375. doi: 10.3389/fonc.2019.01375 (PMC6927466; doi:10.3389/fonc.2019.01375)
Supplement: Supplementary file 1 [file Data_Sheet_1.docx]

Supplementary Table 1

**Genomic mutation analysis**

The clinical sequencing system at the Hokkaido University Hospital for cancer precision medicine was previously reported. (Hayashi H, et al. Pancreatology 2018 18, 647-58)

Oncogene mutation:

Major mutation (1) TP53 Q104* (25.70%)

Minor mutation (2) BRCA1 E1754G (15.20%)

SNP: TP53 P72R (100.00%)

VUS: ARID2 (3.20%), MLH1 (4.00%)

CNV (CN>=4 or CN <= 1.4): 47

Amplified: N/A

Reduced: TNFRSF14, SDHB, ARID1A, MUTYH, DNMT3A, VHL, BAP1, GATA2

TERT, CSF1R, FANCE, CARD11, SMO, PAX5, ABL1, RET, SUFU, MEN1, KMT2D

PTPN11, HNF1A, SLC7A8, AKT1, MAP2K1, IDH2, TSC2, CREBBP, CDH1

FANCA, TP53, FLCN, ERBB2, CD79B, STK11, MAP2K2, SMARCA4, JAK3, AKT2

CD79A, CIC, SRC, SMARCB1, CHEK2, NF2, CRLF2, AMER1, MED12

Fusion: N/A
