# Supplementary material for: Lymph Node Metastasis From Gastroesophageal Cancer Successfully Treated by Nivolumab: A Case Report of a Young Patient
Source: Front Oncol. 2019 Dec 16;9:1375. doi: 10.3389/fonc.2019.01375 (PMC6927466; doi:10.3389/fonc.2019.01375)

Supplementary file 1

**Histological analysis**

Histological results of H&E staining and immunohistochemiscal staining of the surgically resected specimen. Gastroesophageal junction carcinoma was diagnosed as poorly differentiated carcinoma with massive lymph node metastasis. The primary tumor and the #16 lymph node metastatic lesion were evaluated by immunohistochemistry.

Lymphocytes were stained by subtype markers, such as CD4 (1F6, Leica Microsystems), CD8 (C8/144B, Agilent Technology), FoxP3 (236A/E7, abcam), and PD-1 (NAT-105, LSBio).

Mismatch repair proteins in the cancer cells were assessed by immunohistochemistry with antibodies for mutL homolog 1 (MLH1), mutS homologue 2 (MSH2), mutS homologue 6 (MSH6), and postmeiotic segregation increase 2 (PMS2). The following antibodies were used: M1 (Roche Diagnostics), G210-1129 (Roche Diagnostics), PU29 (Leica Microsystems), and EP52 (Agilent Technology) for MLH1, MSH2, MSH6, and PMS2, respectively. The PD-L1 antibody was E1L3N (Cell Signaling Technology).

Epstein-Barr virus (EBV)-encoded RNA *in situ* hybridization was incubated with BOND EBER Probe (Leica Microsystems, Welzlar, Germany). The reaction was detected with BOND Polymer Refine Detection using a Bond-III fully automated stainer (Leica Biosystems, Nussloch, Germany).

These analyses were approved by Asahikawa Medical University Ethics Committee (No 18261).

Figure S1A. A histological analysis of the gastroesophageal junction carcinoma. Massive tumor infiltrating lymphocytes were not observed and lymphocytes were not specifically stained for subtype markers (e.g., CD4, CD8, FoxP3, or PD-1) (Original magnification x40). The decreased expression of mismatch repair proteins was not observed in the cancer cells, resulting in mismatch-repair proficient (MMR-p). The gastric cancer cells were negative for PD-L1 (Original magnification x200), but the membrane of the cancer cells was positive for PD-1. EBER in situ hybridization was negative.

Figure S1B. The histological analysis of the metastatic lymph node. CD4-positive lymphocytes were predominant in comparison to CD8-positive lymphocyte. The staining patterns were similar to those of the primary lesion. Both cancer cells and stromal cells were negative for PD-L1.

Figure S1A

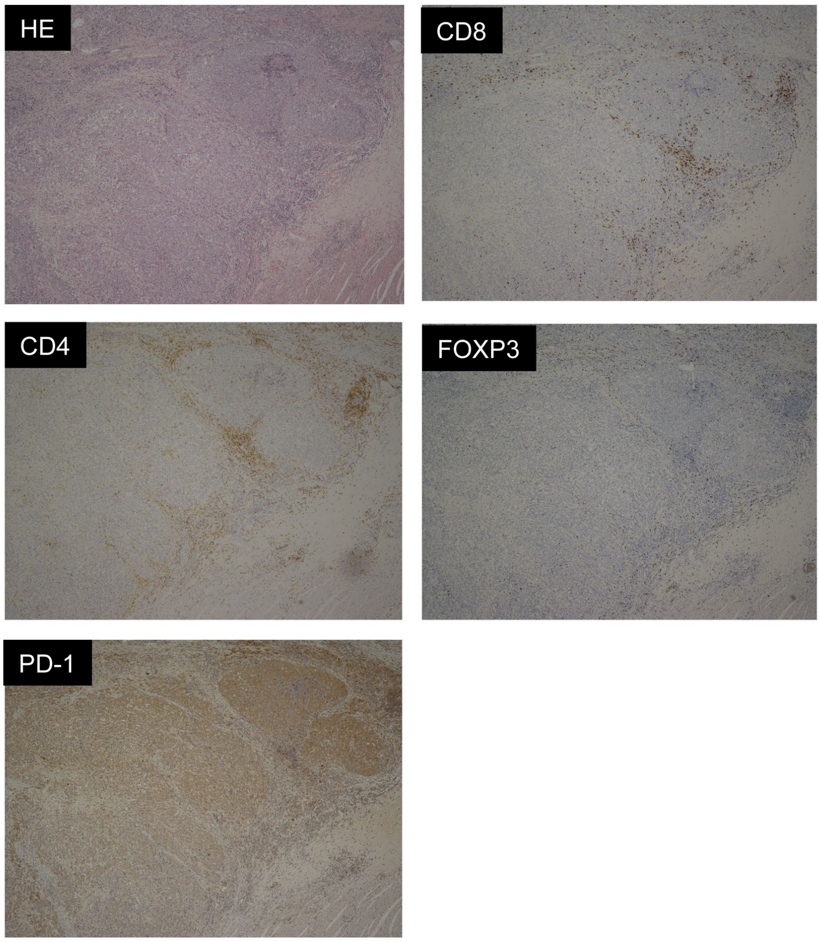

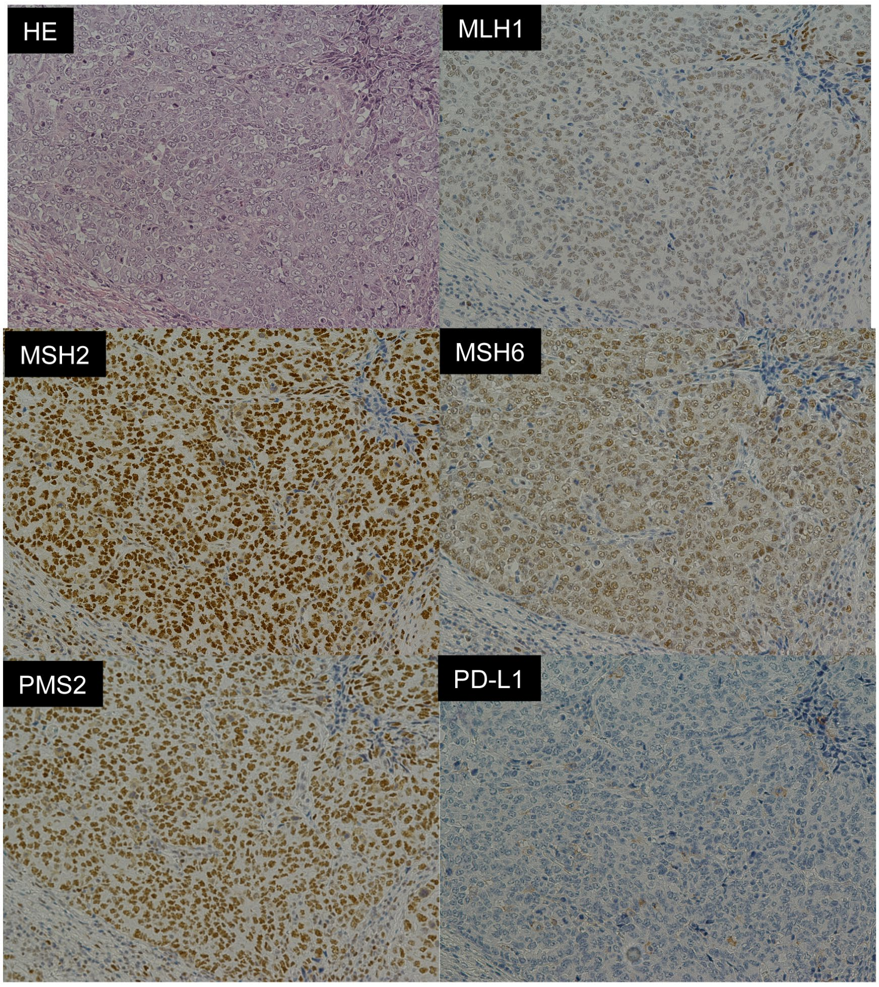
HE
MLH1
MSH2
MSH6
PMS2
PD-L1

EBER-ISH

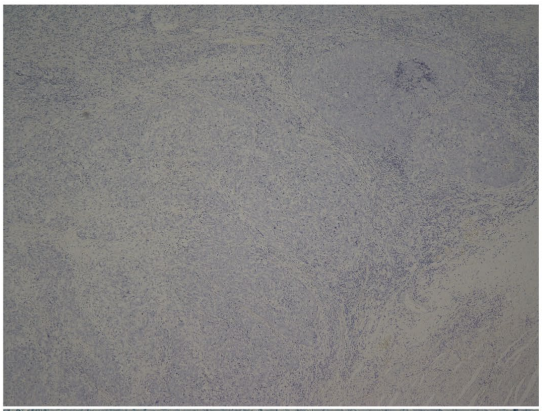

x40

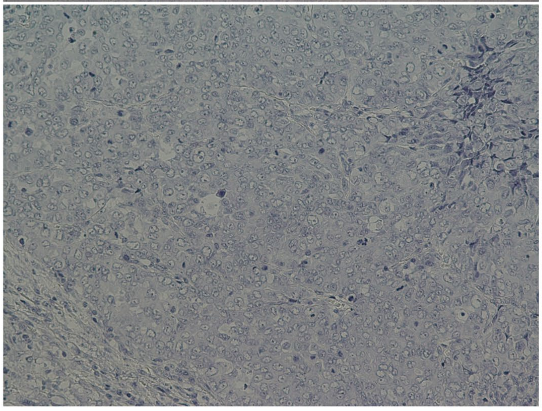

x200

Figure S1B

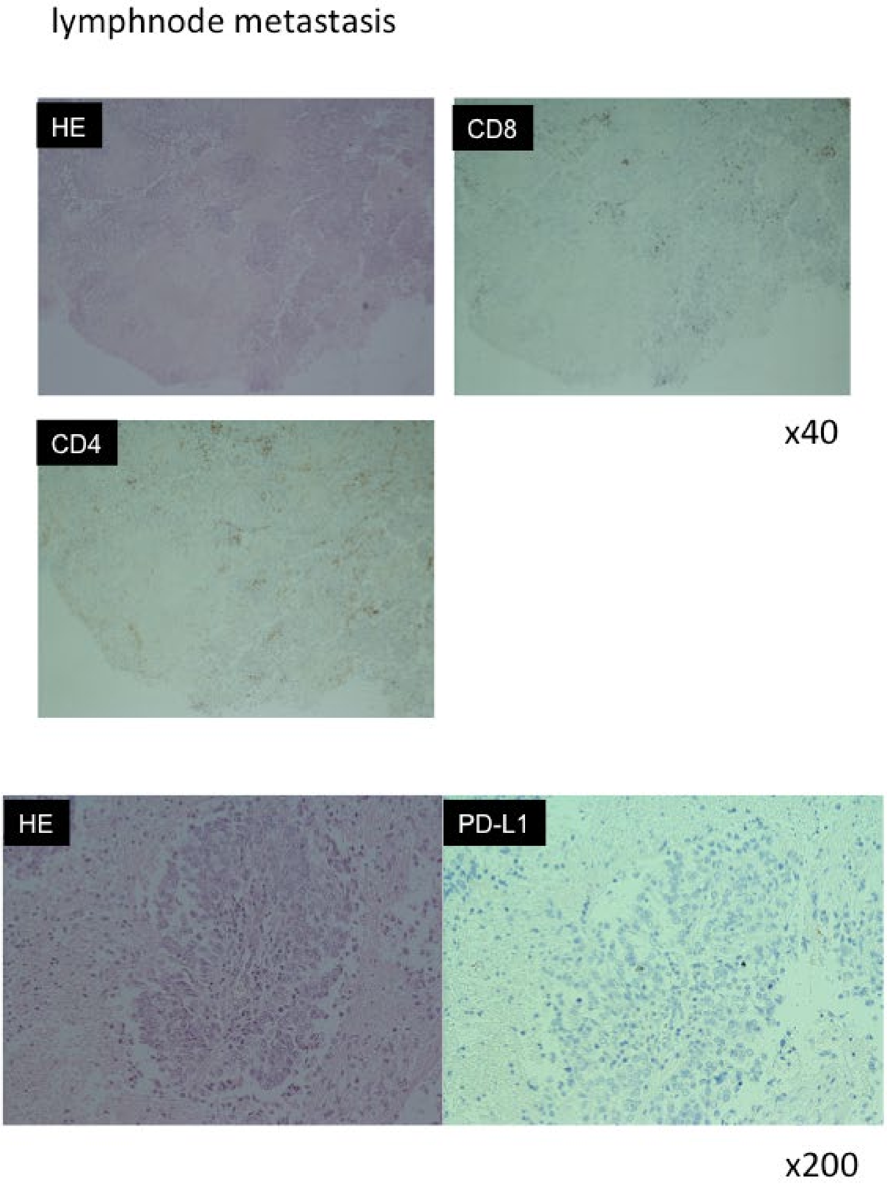

Supplement: Supplementary file 2 [file Image_1.pdf]
