# Supplementary material for: Lymph Node Metastasis From Gastroesophageal Cancer Successfully Treated by Nivolumab: A Case Report of a Young Patient
Source: Front Oncol. 2019 Dec 16;9:1375. doi: 10.3389/fonc.2019.01375 (PMC6927466; doi:10.3389/fonc.2019.01375)

Supplementary file 2

Microsatellite instability analysis

DNA was purified from Formalin fixed paraformaldehyde embedded sample of gastric cancer and normal tissue using Gene Read FFPE kit (Qiagen). Polymerase chain reactions were amplified with the microsatellite markers, BAT-26, NR-21, BAT-25, MONO-27, and NR-24. Capillary electrophoresis was performed with Compact CE Sequencer (Hitachi high-Tech, Osaka, Japan) and DNA fragment analysis were reported with Gene Marker software (SoftGenetics, PA).

Figure S2. There was no shift in the peak of microsatellite markers on comparing the cancerous (blue and green) and normal tissue (red), indicating microsatellite stability (MSS).

Figure S2.

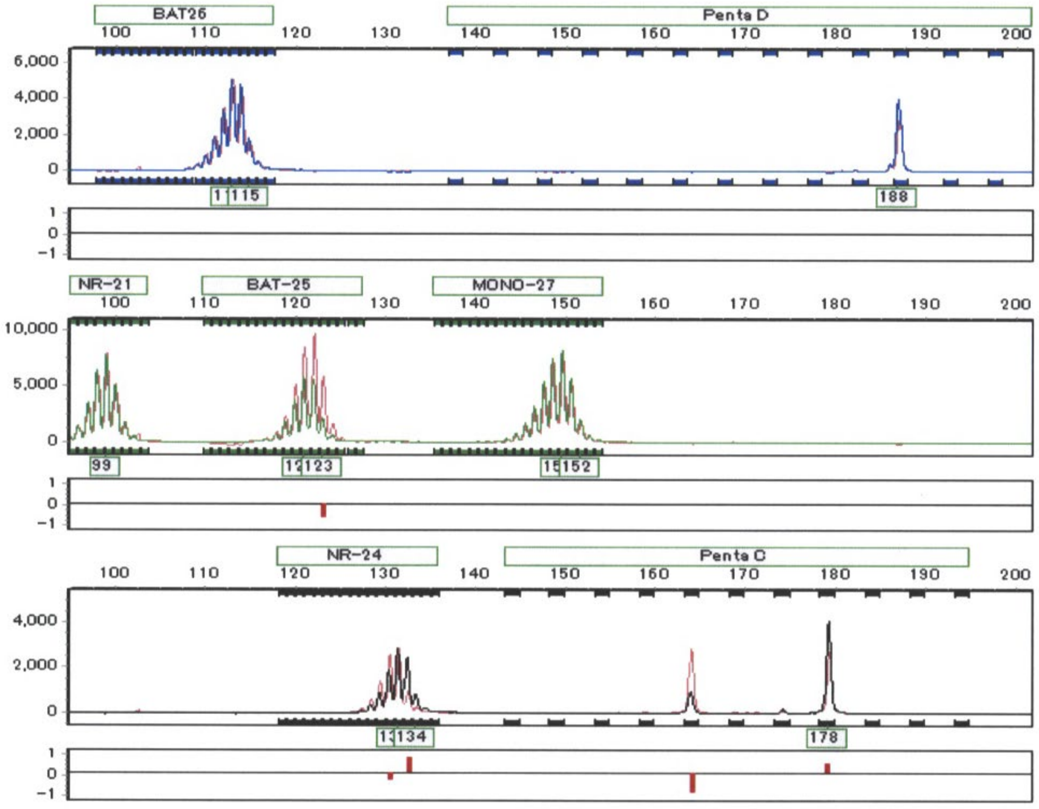

Supplement: Supplementary file 3 [file Image_2.pdf]
